# Hyperventilation strain CMR imaging in patients with acute chest pain

**DOI:** 10.1038/s41598-022-17856-y

**Published:** 2022-08-09

**Authors:** Deborah Siry, Johannes H. Riffel, Janek Salatzki, Florian Andre, Marco Ochs, Lukas D. Weberling, Evangelos Giannitsis, Hugo A. Katus, Matthias G. Friedrich

**Affiliations:** 1 Department of Cardiology, Angiology and Pneumology, University of Heidelberg, Heidelberg, Germany; 2 Department of Cardiology and Angiology, Robert-Bosch-Hospital, Stuttgart, Germany; 3 Department of Cardiology, Angiology and Internal Intensive Care, Theresien-Hospital, Mannheim, Germany; 4 Departments of Medicine and Diagnostic Radiology, McGill University Health Centre, 1001 Decarie Blvd, Montreal, QC H4A 3J1 Canada; 5 DZHK (German Centre for Cardiovascular Research), Partner Site Heidelberg, Heidelberg, Germany

**Keywords:** Cardiology, Medical research

## Abstract

In patients with suspected acute coronary syndrome high-sensitivity cardiac tropnonin T is used for rapid patient triage. Some acute coronary syndrome patients assigned to the observe zone based on high-sensitivity cardiac troponin T after 1 h require further diagnostic testing. Fast-strain encoded CMR imaging with breathing maneuvers may accelerate diagnostic work-up and identify patients suffering from acute coronary syndrome. Patients presenting with acute chest pain (high-sensitivity cardiac troponin T level 5–52 ng/L) were prospectively enrolled (consecutive sampling, time of recruitment: 09/18–06/19). Fast-strain-encoded imaging was performed within the 1-h timeframe (0 h/1 h algorithm) prior to 2nd high-sensitivity troponin T lab results. Images were acquired at rest as well as after 1-min of hyperventilation followed by a short breath-hold. In 108 patients (59 male; mean age: 57 ± 17y) the mean study time was 17 ± 3 min. An abnormal strain response after the breathing maneuver (persistent/increased/new onset of increased strain rates) correctly identified all 17 patients with a high-sensitivity troponin T dynamic (0 h/1 h algorithm) and explanatory significant coronary lesions, while in 86 patients without serologic or angiographic evidence for severe coronary artery disease the strain response was normal (sensitivity 100%, specificity 94.5%; 5 false positive results). The number of dysfunctional segments (strain > − 10%) proved to be a quantifiable marker for identifying patients with acute coronary syndrome. In patients with suspected acute coronary syndrome and inconclusive initial high-sensitivity troponin T, fast-strain-encoded imaging with a breathing maneuver may safely and rapidly identify patients with acute coronary syndrome, without the need for vasodilators, stress, or contrast agents.

## Introduction

Chest pain is one of the most common symptoms in patients presenting to emergency departments (ED). Among various differential diagnoses to be considered, acute coronary syndrome (ACS) is critical because of the potential need for an immediate therapeutic intervention^1^.

According to current guidelines, patients with suspected ACS should undergo an electrocardiogram (ECG) within 10 min, as well as serial biomarker measurements such as high-sensitivity cardiac troponin T (hscTnT)^2^. Recently, a new generation of hscTnT assays—the so-called 0 h/1 h algorithm—has been adopted in triaging patients with suspected NSTEMI (non-ST-elevation myocardial infarction) within one hour^3^. However, not all patients can be safely classified after 1 h: By means of the 0 h/1 h protocol, about 15% of patients require further observation^4,5^. About one third of patients initially assigned to the “observe” zone are eventually diagnosed with NSTEMI. While waiting for further diagnostic tests, patients with actual ischemia in the “observe” zone may suffer from progressive myocardial injury. In fact, recent data indicate that patients allocated to the “observe-zone” may have a higher long-term mortality rate than patients initially ruled-in according to hscTnT kinetics^6^.

Despite its unique tissue characterization capabilities and comprehensive range of quantitative markers, cardiovascular magnetic resonance (CMR) imaging is rarely used in acute clinical settings, mainly due to the associated logistical effort, lengthy protocols, and the frequent need for contrast agents.

Compared to standard CMR protocols, fast Strain-encoded Imaging (fSENC) is an ultrafast (single-heartbeat) method for quantifying myocardial strain, a sensitive marker for abnormal regional and global ventricular function^7,8^. After manual contouring, a color-coded strain map is produced that reflects strain values on a pixel level^9^. In recent studies, fSENC has proven to be highly reproducible^10^. Furthermore, it allows for reliable differentiation between subendocardial and transmural myocardial infarction^11^. We could recently demonstrate the feasibility of fSENC imaging in patients presenting with new onset of chest pain^12^.

Hyperventilation leads to transient reduction of coronary blood flow due to the associated reduction of blood carbon dioxide. Accordingly, hyperventilation has been used for diagnosis of myocardial ischemia^13^. Recently, oxygenation-sensitive CMR has been evaluated during a voluntary breath-hold after hyperventilation to identify coronary vascular dysfunction^14^. While in healthy subjects, hyperventilation leads to coronary vasoconstriction and breath-holding triggers coronary vasodilation, territories with abnormal coronary vascular function have a blunted or even absent vasomotor response^14,15^. Breath-hold after hyperventilation may lead to a more pronounced vasodilation and subsequent increase in myocardial oxygenation than intravenous adenosine infusion^14–16^. Protocols with a hyperventilation breath-hold (HVBH) have already been safely applied in patients with coronary artery disease (CAD)^17,18^.

## Methods

### Study population

In a double-blinded study design, patients with acute chest pain suggestive of ACS presenting at our chest pain unit were recruited. Other results of this study cohort have been previously reported^12^.

All patients with an initial hscTnT between 5 and 52 ng/L on admission (0 h/1 h algorithm) underwent a focused fSENC-CMR scan within 1 h after first hscTnT measurement (before second). Patients were closely monitored (ECG, pulse oximetry), and accompanied by a physician during in-hospital transport and during the CMR scan^2^ (Table 1). The study was approved by the local ethics committee (Ethikkommission Medizinische Fakultät Heidelberg (S-483/2018)). All participants provided informed written consent. The study was performed in accordance with relevant guidelines and regulations.

**Table 1:** Inclusion and exclusion criteria of the study^30^.

Inclusion criteria	Exclusion criteria
Acute chest pain HEART score ≤ 6 Initial hscTnT 5–52 ng/L Signed informed consent	Acute ST-elevation myocardial infarction Hemodynamic instability Cardiogenic shock Mechanical complications of MI Systolic heart failure (LVEF < 40%) Life-threatening arrhythmias History of CAD CMR: non-suitable metallic implants CMR: severe claustrophobia

*hscTNT* high-sensitive cardiac troponin T, *LVEF* left ventricular ejection fraction, *CAD* coronary artery disease, *MI* myocardial infarction, *CMR* cardiovascular magnetic resonance.

### CMR examination

CMR scans were performed in a 1.5 T whole-body CMR scanner (Ingenia CX 1.5 T, Philips Medical Systems, Best, The Netherlands) or, in 16 individuals, in a 3 T whole-body scanner (Ingenia CX 3T, Philips Medical Systems, Best, The Netherlands). The images were displayed in real-time on a computer screen. Patients were in direct contact with the physicians and followed breathing commands through the built-in audio system.

A vector ECG was used for R-wave triggering. Cine images were acquired using a standard cine sequence in 35 acquired phases covering the whole left ventricle from base to apex, as well as in a 2-chamber view and a 4-chamber view (Field of view 140 mm^2^, TE 1.38 ms, TR 2.77 ms, flip angle 60°, pixel size 0.88 × 0.88 mm^2^, 35 acquired phases, slice thickness 8 mm). K space was filled through spiral acquisition. The fSENC images were planned on an end-systolic timeframe centered on the LV, i.e. 3 long axis views (2-chamber view, 3-chamber view, and 4-chamber view) and 3 short axis views (basal, midventricular, apical).

**Figure 1:**
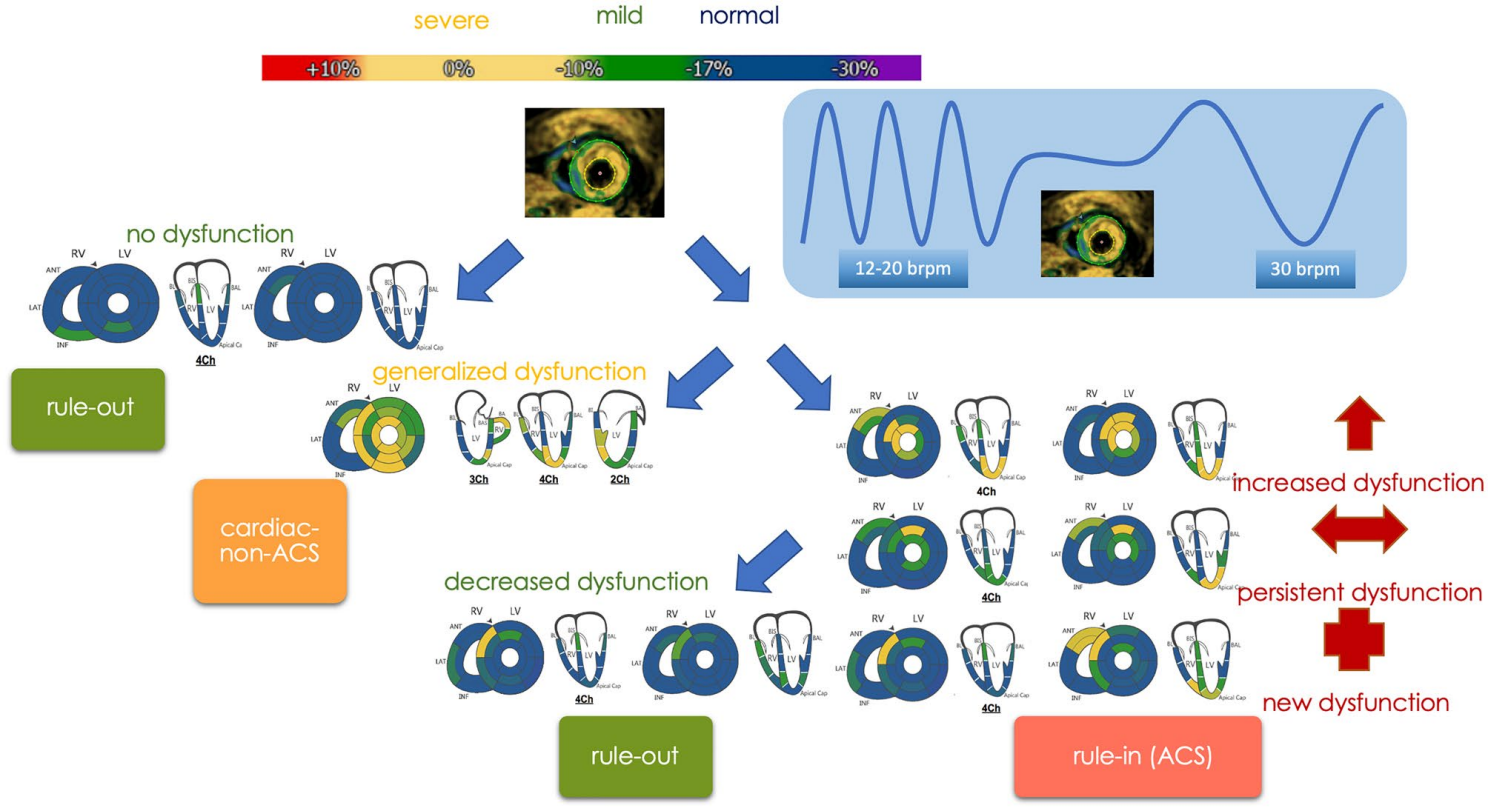
Analysis workflow: triage according to change in myocardial deformation before (left) and after hyperventilation: 1 min at 30 brpm followed by a short breath-hold for image acquisition (right). Rule-out = normal contractility at rest and after hyperventilation or improved contractility after hyperventilation; cardiac, non-ACS = generally impaired myocardial deformation; rule-in = increased/persistent or new onset of dysfunctional segments after hyperventilation.

Additional fSENC images in a 4-chamber view and 3 short axis views were acquired directly after HVBH. (Field of view 100^2^ mm, TE 0.71 ms, TR 12.16 ms, flip angle 30°, pixel size 1 × 1 mm^2^, slice thickness 10 mm) (Fig. 1).

### Reference standard

The standard of reference was based on the patient’s final clinical diagnosis as determined by staff cardiologists blinded to the CMR results, according to serial hscTnT testing and, if clinically indicated, further diagnostic procedures (coronary angiography, echocardiography, stress ECG, stress CMR or coronary CT). For hscTnT measurements, a 4^th^ generation cTnT assay was used (Roche Diagnostics, Penzberg, Germany)^19^.

If patients were transferred for coronary angiography, significant stenoses were defined as a visual stenosis ≥ 70% of the coronary diameter.

### Image analysis

For strain measurements, we used the certified software “MyoStrain” (Myocardial Solutions Inc., Morrisville, NC, USA). The analyses were performed by a MyoStrain-certified examiner blinded to the 2nd hscTnT results.

Endocardial and epicardial borders were contoured manually. Papillary muscles, trabeculae and epicardial fat were excluded from the blood pool.

The circumferential and longitudinal strain values were represented in a color-coded image according to the AHA (American Heart Association) 16-segment model, based on previously established cut-off values^20^:

- Normal myocardial deformation (strain < − 17)
- Mild reduction of myocardial deformation (strain > − 17)
- Severe reduction of myocardial deformation (strain > − 10)

By visual assessment of the fSENC bull’s-eye maps, patients were triaged either to 0: non-cardiac, 1: ACS or 2: cardiac, non-ACS causes of acute chest pain. Maps devoid of dysfunctional segments (strain > − 10) after HVBH were considered normal and classified to group 0, whereas patients with persistent, increased, or new onset of regional dysfunction after HVBH were classified to group 1, and patients with global dysfunction not compatible with coronary artery territories were assigned to group 2 (Fig. 1).

Within group 1, the dysfunctional segments were allocated to specific coronary artery territories according to AHA recommendations^21^.

### Statistics

The primary endpoint of this study was defined as the diagnostic accuracy of fSENC and HVBH in predicting ACS (H_0_: diagnostic accuracy of fSENC + HVBH < 80% compared to the gold standard). We calculated a sample size of 108 patients (diagnostic accuracy > 90%, power 80%, *p* value < 5%).

All statistical analyses were performed using the computer programs Microsoft Excel (Microsoft, Redmond, CA, USA), SPSS (Version 24, IBM, Armonk, USA) and MedCalc (Version 19.2, MedCalc Software, Ostend, Belgium).

Quantitative data included mean values as well as standard deviation (SD). Receiver Operating Characteristic (ROC) curves and logistic regression curves were compared using the Hanley and McNeil test^22^.

A Kolmogorov–Smirnov test (2-sided) for random distribution, McNemar’s test (2-sided) to test fSENC results for deviation from the gold standard and Mann–Whitney U test (2-sided) to detect differences between diagnostic performance of fSENC ± HVBH and hscTnT as well as between time intervals to treatment were performed. Cohen’s Kappa coefficient was calculated to evaluate correlation between culprit coronary lesions/severe stenoses as assessed by coronary angiography and fSENC maps. The intraclass correlation coefficient (ICC) (2-sided) was performed to assess interobserver reliability. P-values < 0.05 were regarded as statistically significant.

### Ethics approval and consent to participate

The study was approved by the local ethics committee (Ethikkommission Medizinische Fakultät Heidelberg (S-483/2018)). All participants provided informed written consent.

## Results

### Study population

We consecutively enrolled a total of 108 patients (49 females, mean age: 57 ± 17y). Of these, 85 were ruled out for a cardiac cause of the chest pain (group 0)—within the rule-out group some patients received additional non-invasive testing as part of routing diagnostic work-up (Echocardiography n = 6, stress-ECG n = 7) with altogether normal results. Another 6 were diagnosed with an underlying cardiac disease (group 2: n = 3 Hypertrophic Cardiomyopathy, n = 1 Dilated Cardiomyopathy, n = 1 Myocarditis, n = 1 Pulmonary Hypertension), whereas 17 were diagnosed with ACS (group 1), including 8 with NSTEMI. Table 2 depicts patient characteristics.

**Table 2:** Patient characteristics.

Total patient population: 108	Count	Mean (± SD)	max/min	Median	IQR
**Sex**					
Female	49 (45%)				
Male	59 (55%)				
Age (years)		57 ± 17	85/20		
BMI (kg/m^2^)		26.6 ± 5.3	52.9/14.8		
BP (systolic) (mmHg)		155 ± 20	204/110		
HR (bpm)		71 ± 16	133/32		
**HEART score**					
Low		41			
Intermediate		67			
**NYHA**					
1	74 (69%)				
2	18 (17%)				
3	15 (14%)				
4	1 (1%)				
EF (%)		65.4 ± 12.9	96.0/20.5	66.4	16.5
EDV (ml)		92.8 ± 39.4	237.2/40.3	79.1	52.5
ESV (ml)		32.5 ± 21.5	132.6/4.5	26.5	18.8
cvRF		2	5/0		
Diabetes	9 (8%)				
Hypertension	58 (54%)				
Hypercholesterinemia	34 (31%)				
Familial predisposition	31 (29%)				
**Nicotine (py)**					
Non-smoker	62 (57%)	0 ± 1	9/0		
Past smoker	32 (30%)	19 ± 15	45/1		
Smoker	14 (13%)	25 ± 20	60/3		
Chest pain duration (h) (ACS)		15.6	> 24/1	24	21
Chest pain duration (h) (cardiac, non-ACS)		15	> 24/0.5	22	20
Chest pain duration (h) (non-cardiac)		14.9	> 24/1	16	19
hscTnT 0 h (ng/L)		11 ± 8	49/5		
hscTnT 1 h (ng/L)		15 ± 21	112/3		
Δ hscTnT (kinetics) (ng/L)		5 ± 18	+ 93/− 28		
**Diagnostic procedures**					
Stress ECG	7 (6%)				
Echocardiography	6 (6%)				
Standard CMR	1 (1%)				
CT angiography	1 (1%)				
Coronary angiography	25 (23%)				

*max* maximum, *min* minimum, *SD* standard deviation *IQR* interquartile range, *BMI* body mass index, *BP* blood pressure, *HR* heart rate, *NYHA* New York Heart Association, *EF* ejection fraction, *ESV* end-systolic volume, *EDV* end-diastolic volume, *cvRF* cardiovascular risk factors, *py* pack years, *h* hours, *ACS* acute coronary syndrome, *hscTNT* high-sensitive cardiac troponin T, *ECG* electrocardiogram, *CMR* cardiovascular magnetic resonance, *CT* computed tomography.

All CMR scans were performed shortly after patient admission with a total mean scan time of 17 ± 3 min (HVBH including instructions: 3 min). In 8 patients, the fSENC images after hyperventilation were not evaluated: 3 of them (group 0 n = 2; group 2 n = 1) were unable to perform the HVBH; in 5 patients (group 1 n = 2; group 0 n = 3), the images were excluded due to low image quality. Therefore, in these 8 patients results were based on the rest images alone.

### Qualitative fSENC analysis

First, fSENC images obtained at baseline (excluding images after HVBH) were analyzed separately and compared to final clinical diagnosis (standard of reference). With 12 false positive and 3 false negative results, an accuracy of 86.1% was achieved. In a separate analysis, both, baseline and HBHV results were evaluated. This resulted in the detection of an additional 3 patients with ACS who developed changes in myocardial strain, while in 7 other patients, hyperventilation was associated with augmented contraction. Therefore, the addition of the HVBH as a stress test improved the diagnostic performance of fSENC to an accuracy of 95.4% (Table 3).

**Table 3:** Diagnosis according to fSENC at rest and after HVBH as compared to reference standard.

	fSENC +	fSENC −	fSENC-HV +	fSENC-HV −	Reference standard
ACS (1)	**14**	**3**	**17**	**0**	**17**
**Non-cardiac (0)/cardiac, non-ACS (2)**					
0	12	65	5	71	85
2	0	14	0	15	6
0 + 2	**12**	**79**	**5**	**86**	**91**
	**26**	**82**	**22**	**86**	**108**

Significant values are in bold.

### Quantitative analysis of fSENC maps

**Figure 2:**
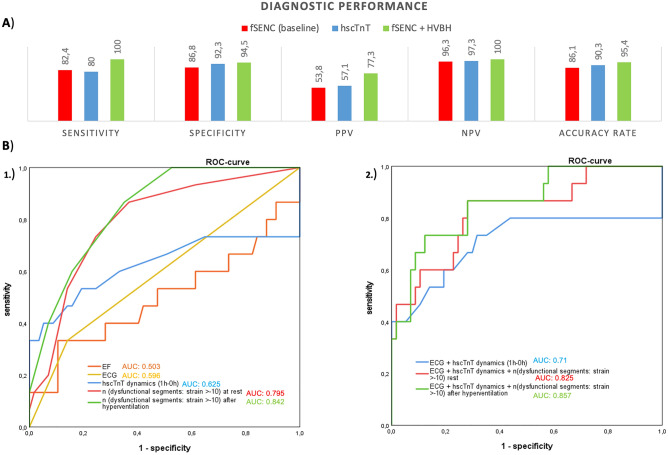
Central illustration of results. (**A**) Comparison of diagnostic performance of fSENC (at baseline) to hscTnT dynamics (1 h–0 h) and fSENC with HVBH. (**B**) ROC curves for ACS identification according to quantifiable markers (EF, ECG (ST-wave abnormalities), hscTnT dynamics, number of dysfunctional segments (strain > − 10) at rest and after hyperventilation) and Logistic regression analysis of combined diagnostic methods: (1) EF (AUC = 0.503; 95% CI 0.31–0.70, *p* = 0.976), ECG (AUC = 0.596; 95% CI 0.43–0.77, *p* = 0.271), hscTnT dynamics (1 h–0 h) (AUC = 0.625; 95% CI 0.41–0.84, *p* = 0.245), number of dysfunctional segments (strain > − 10) at rest (AUC = 0.795; 95% CI 0.67–0.92, *p* < 0.0001) and number of dysfunctional segments after hyperventilation (AUC = 0.842; 95% CI 0.75–0.94, *p* < 0.0001). Comparison of ROC curves: EF vs. n(ds rest): 95% CI 0.07–0.51, *p* < 0.01; EF vs. n(ds hv): 95% CI 0.13–0.55, *p* < 0.002; ECG vs. n(ds rest): 95% CI 0.0003–0.40, *p* < 0.05; ECG vs. n(ds hv): 95% CI 0.06–0.43, *p* < 0.01); hscTnT vs. n(ds rest): 95% CI − 0.07 to 0.41, *p* = 0.158; hscTnT vs. n(ds hv): 95% CI − 0.0004 to 0.44, *p* < 0.05; n(ds rest) vs. n(ds hv): 95% CI − 0.07 to 0.17, *p* = 0.446. (2) AUC: routine clinical diagnostic work-up (ECG + hscTnT dynamics) = 0.71 (95% CI 0.52–0.90, *p* < 0.035). AUC after adding the number of dysfunctional segments (strain > − 10) at rest: AUC = 0.825; 95% CI 0.70–0.95, *p* < 0.0001/after hyperventilation: AUC = 0.857; 95% CI 0.75–0.96, *p* < 0.0001. Comparison of ROC curves: ECG + hscTnT vs. ECG + hscTnT + n(ds rest): 95% CI − 0.02 to 0.25, *p* = 0.099; ECG + hscTnT vs. ECG + hscTnT + n(ds rest) + n(ds hv): 95% CI − 0.02 to 0.31, *p* = 0.079.

For quantitative assessment of myocardial strain, the number of dysfunctional segments (strain > − 10) before and after hyperventilation were plotted in a ROC-curve for ACS identification. Patients with non-ischemic heart disease were excluded. The AUC (area under the curve) for the number of dysfunctional segments (ds) at rest was 0.795 (95% CI 0.67–0.92, *p* < 0.0001), for hscTnT dynamics 0.625 (95% CI 0.41–0.84, *p* = 0.245), and for the number of dysfunctional segments after hyperventilation 0.842 (95% CI 0.75–0.94, *p* < 0.0001). The ROC curves for the number of dysfunctional segments at rest and after hyperventilation were significantly different from those for hscTnT dynamics (hscTnT vs. n(ds rest): 95% CI − 0.07 to 0.41, *p* = 0.158; hscTnT vs. n(ds hv): 95% CI − 0.0004 to 0.44, *p* < 0.05), however did not differ significantly from each other (95% CI − 0.07 to 0.17, *p* = 0.446) The AUC of a routine clinical diagnostic work-up with ECG and hscTnT dynamics (1 h–0 h) (AUC ECG + hscTnT: 0.71; 95% CI 0.52–0.90, *p* < 0.035) was significantly improved when the number of dysfunctional segments (strain > − 10) at rest (AUC ECG + hscTnT + n(ds rest): 0.825; 95% CI 0.70–0.95, *p* < 0.0001) or after hyperventilation (AUC ECG + hscTnT + n(ds rest) + n(ds hv): 0.857; 95% CI 0.75–0.96, *p* < 0.0001) was added (Fig. 2B).

### Qualitative fSENC analysis compared to hscTnT kinetics (0 h/1 h algorithm)

According to the 0 h/1 h algorithm and clinical findings, 14 patients were ruled in for ACS. In 8 of these 14 patients coronary angiography was performed and thereafter discharged. 74 patients were ruled out for acute myocardial injury in accordance with the 0 h/1 h algorithm. In 4 of the 74 patients, invasive coronary angiography was conducted based on clinical presentation and in 2 of these 4 cases, significant CAD was found.

The remaining 20 patients were assigned to the observe zone after 1 h requiring further diagnostic procedures. In 10 of these patients, coronary angiography was performed revealing significant CAD in 7 cases (Tables 4, 5). Therefore, serial hscTnT measurements alone regarding the 88 patients with a definitive diagnosis after 1 h resulted in a sensitivity of 80%, specificity of 92.3% and an accuracy of 90.3%.

**Table 4:** Patient classification according to 0 h/1 h algorithm after 1 h with number of patients who underwent coronary angiography.

	Rule-in	Observe-zone	Rule-out	
0 h/1 h algorithm	14	20	74	108
Final diagnosis: ACS	8	7	2	17
Final diagnosis: non-cardiac/cardiac, non-ACS	6	13	72	91
Coronary angiography	11	10	4	25

*ACS* acute coronary syndrome, *hscTnT* high-sensitive cardiac troponin T.

The combination of fSENC images at rest as well as the images after the HVBH proved to be superior to serial hscTnT measurements (Fig. 2A).

### Observe zone (exploratory subgroup analysis)

In our patient cohort, 35% of the patients classified to the observe zone were found to have significant CAD and, after full clinical diagnostic evaluation, underwent a therapeutic coronary intervention. Assignment to the observe zone resulted in a significant increase of the mean time-to-treatment (coronary intervention) for ACS patients (54.0 ± 37.6 h) in comparison to patients who were deemed “ruled in” based on hscTnT kinetics (8.1 ± 6.8 h) (95% CI 7.5 h–84.8 h; *p* < 0.05). fSENC with hyperventilation correctly diagnosed all 20 patients assigned to the observe zone within the 1-h time frame after admission (group 0 n = 13; group 1 n = 7).

### Culprit coronary lesions (exploratory subgroup analysis)

**Figure 3:**
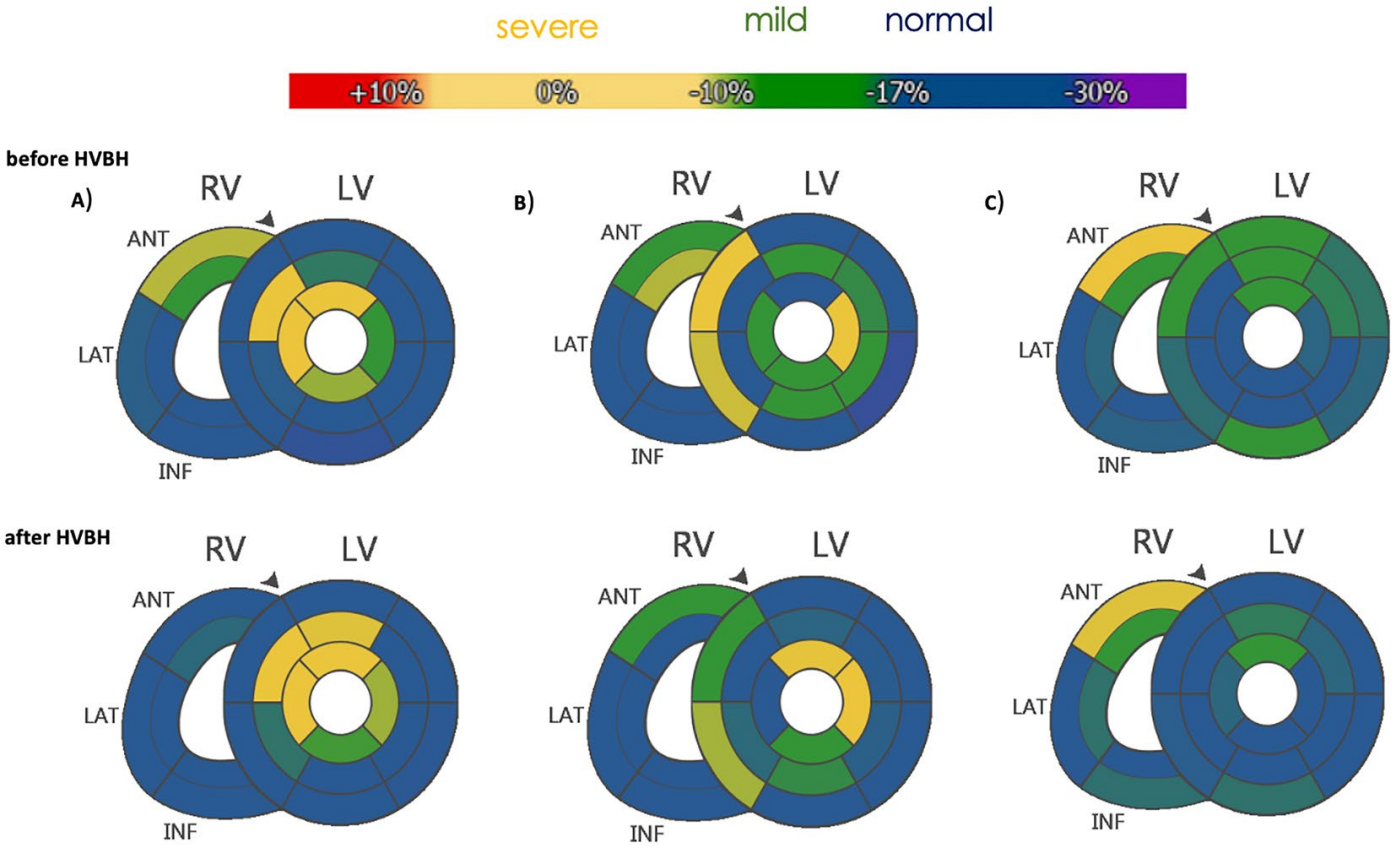
Examples of fSENC bull’s eye plots before HVBH (above) and after HVBH (below). (**A**) LAD stenosis with increased dysfunction after HVBH, (**B**) LCX stenosis with increased dysfunction after HVBH, (**D**) RCA stenosis with persistent dysfunction after HVBH.

The affected coronary artery territories of the 17 patients with ACS were identified visually, using fSENC bull’s eye plots according to the AHA 17-segment model (Fig. 3). In 14 of the 17 patients (82%), the culprit coronary lesions and significant stenoses (stenosis ≥ 70% by visual analysis; target vessels for acute intervention) could be correctly identified by fSENC (Table 5).

**Table 5:** Culprit coronary lesions/significant stenoses as identified visually by fSENC bulls-eyes in comparison to coronary angiography results.

	Coronary angiography				
	LAD	RCA	LCX	Multiple	Total
**fSENC**					
LAD	4	0	0	0	4
RCA	0	1	1	0	2
LCX	0	0	1	0	1
Multiple	2	0	0	8	10
Total	6	1	2	8	17

*LAD* left anterior descending, *RCA* right coronary artery, *LCX* left circumflex.

Cohen’s Kappa coefficient for the agreement between fSENC results and the significant coronary lesions according to coronary angiography was strong (0.718; *p* < 0.05).

**Figure 4:**
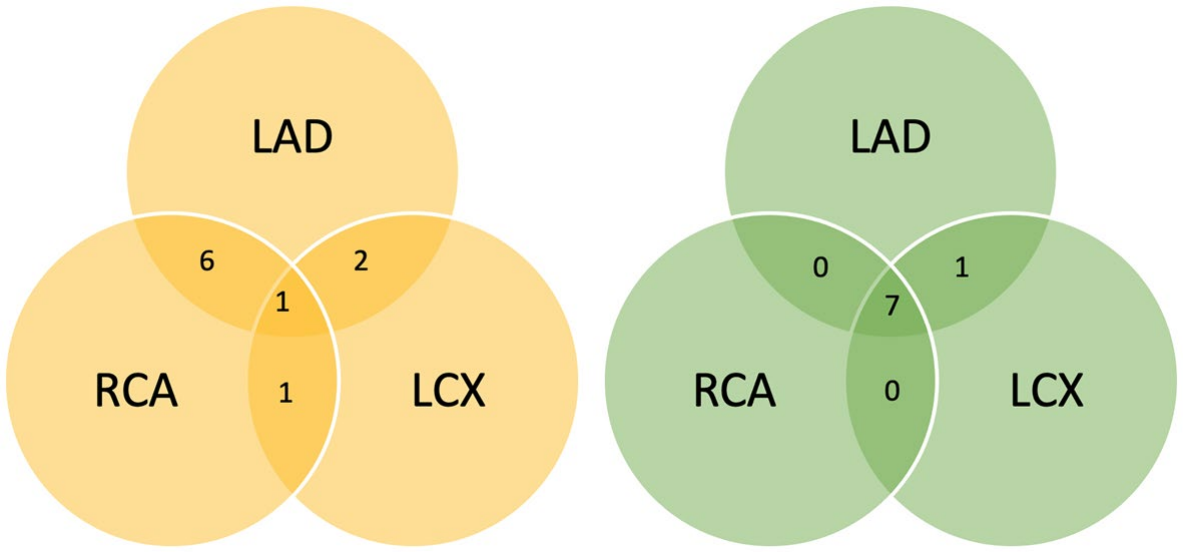
Venn diagram of the 8 multi-vessel-disease cases as defined by fSENC (left) and coronary angiography/bypass operation (right).

In 6 of the 8 patients with multi-vessel disease fSENC showed a combination of LAD (left anterior descending) and RCA (right coronary artery) stenoses. LCX (left circumflex) stenoses > 70% in three-vessel disease (according to coronary angiography/bypass operation) were not well detected by fSENC (Fig. 4).

### Interobserver Reliability

Fifteen patients were analyzed by a second certified MyoStrain reader blinded to all patient data and previous fSENC results. An ICC of 0.96 and 0.96 was reached for the number of dysfunctional segments (strain > − 10) and the number of segments with a strain > − 17 respectively. These results demonstrate a high level of reproducibility (*p* < 0.0001).

## Discussion

To our knowledge, this is the first study to assess the diagnostic performance of CMR strain imaging using a standardized breathing maneuver as a replacement for physical or pharmacological stress in patients with acute chest pain. The main findings of our study are:

1. The diagnostic performance of a visual evaluation of fSENC maps before and after HVBH was very high (accuracy 95.4%). In all but 5 patients, an ischemic cause of the acute chest pain could be correctly ruled in or ruled out. There were no false negative results.
2. The number of dysfunctional segments before and after hyperventilation may serve as a quantifiable surrogate marker for ischemic burden.
3. fSENC and HVBH correctly diagnosed all 20 patients within one hour who were assigned to the observe zone according to the hscTnT 0 h/1 h algorithm.
4. Additionally, visual analysis of the fSENC images accurately identified the culprit coronary artery and relevant stenoses in most patients.

Our results may have significant clinical implications. As early revascularization in NSTEMI patients improves patient prognosis compared to delayed invasive strategy^1^, an accelerated diagnostic workup would shorten time to treatment and thus have a positive impact on patient outcome. Early rule-out diagnostic tests such as fSENC may significantly reduce staff time and save hospital costs by shortening the length of stay in the CPU and reducing procedural costs^23^.

In a recent study we could demonstrate the feasibility of fSENC for diagnostic triage of patients presenting with acute chest pain. Global strain measurements allowed for a safe identification of obstructive CAD—even outperforming ECG and serial hscTnT measurements^12^. Although fSENC images at rest achieved a good diagnostic performance when compared to serial hscTnT measurements in our study, the added input of the images after hyperventilation improved diagnostic performance, especially regarding sensitivity. Major advantages of the protocol are its safety, completely non-invasive setup, the lack of radiation exposure, and its low rate of only minor, transient adverse effects^17^. Confirming previous studies, hyperventilation was well tolerated in our study, despite consisting of a patient population with acute/recent onset of symptoms. Only 3 out of the 108 patients were unable to perform the hyperventilation maneuver.

In a recent study conducted by Fischer et al., it could be shown that Oxygenation-Sensitive CMR in combination with hyperventilation allows for the detection of regional myocardial abnormalities related to multi-vessel CAD without the need of medication or contrast agent^17^. Accordingly, in our study we combined the HVBH with strain imaging and received similar results thereby underlining the clinical impact of the HVBH as a non-invasive stress test in combination with novel emerging imaging techniques.

Ochs et al. compared standard adenosine perfusion stress CMR to strain imaging after adenosine infusion as well as strain imaging after hyperventilation. Both, adenosine-strain and HVBH-strain were found to be superior to standard adenosine first-pass perfusion for identification of obstructive CAD^18^. Our data demonstrate that this approach is safe and applicable in an acute setting as well.

Of particular importance, patients assigned to the observe zone may have a specific additional benefit from fSENC-CMR and HVBH. In studies on the utility of 1-h protocols, the percentage of patients assigned to the observe-zone varied between 30.5%^24^ and 33%^25^, effectively leaving one third of all patients with thoracic pain in the ED without a clear diagnosis after one hour. Therefore, appropriate treatment often is delayed, negatively affecting patient outcome, wait times, and costs. Studies have shown a relatively high prevalence of myocardial infarction in the observe zone group^26,27^. In our study population the prevalence of ischemia within the observe zone was 35%. Of note, the mean time-to-treatment (coronary intervention) of the 7 patients within the observe zone diagnosed with ischemia was 54.0 ± 37.6 h—significantly higher than the 8.1 ± 6.8 h required for patients with positive hscTnT kinetics (rule-in according to 0 h/1 h algorithm).

Additionally, of the 20 patients classified to the observe zone, 10 underwent coronary angiography—3 of whom were discharged thereafter. For these 3 patients, invasive diagnostic procedures including radiation could have been avoided based on hyperventilation CMR strain imaging results alone. Thus, this approach could be of particular value in this high-risk patient group.

Furthermore, hyperventilation CMR strain imaging allowed the correct identification of severe stenoses in most cases. There were inaccuracies with lesions in the LCX territory in patients with three-vessel disease, although variations of RCA and LCX territories are well known and could explain the observed discrepancies^28^. Although coronary angiography remains the gold standard, hyperventilation CMR strain imaging may provide strong clues regarding possible culprit coronary lesions.

Comparatively, strain analysis tools based on analyzing standard cine images such as feature tracking do not require additional sequences and thus allow for shorter scan times which may be useful and efficient. Strain imaging based on cine images however is hampered by through-plane motion artifacts and partial volume effects^29^. We have compared findings by Feature Tracking to fSENC within this patient population. Results will be published shortly.

### Study limitations

We excluded patients with history of PCI/bypass operation and with heart failure (EF < 40%). The study population and the final sample size of those deemed to have ACS was modest and confined to a single center, hence no significant difference within the ROC curve analysis could be registered. Findings need to be confirmed in bigger study population. Additionally, absolute values were not analyzed. Furthermore, CAD was determined by visual assessment of the angiographer, and its hemodynamic relevance was not quantified by Fractional Flow Reserve or instantaneous wave-free ratio. No additional standard stress CMR protocols for comparison to our proposed protocol were performed. CMR as well as fSENC and the relevant expertise is currently not available at all clinical institutions. Additionally, fSENC is at this point still comparatively expensive (one scan: circa 385€ vs. one hscTnT test: circa 2,50€).

## Conclusions

Our suggested protocol achieved a high diagnostic accuracy, outperforming other clinical markers for ACS identification. This approach may be particularly useful for “troponin observe zone” patients, allowing for a safe, drug-free, and non-invasive assessment of myocardial function.

## Data Availability

The datasets used and/or analysed during the current study are available from the corresponding author on reasonable request.

## Abbreviations

CMR: Cardiovascular magnetic resonance
fSENC: Fast Strain ENCoded
HVBH: Hyperventilation breath-hold
ACS: Acute coronary syndrome
hscTnT: High-sensitive Troponin T
GCS: Global circumferential strain
GLS: Global longitudinal strain
ECG: Electrocardiogram
BRPM: Breaths per minute
ICC: Intraclass correlation coefficient
AUC: Area under the curve
ED: Emergency department
ROC: Receiver Operating Characteristic
CAD: Coronary artery disease
NSTEMI: Non-ST-elevation myocardial infarction
EF: Ejection fraction
ESV: End-systolic volume
EDV: End-diastolic volume
LAD: Left anterior descending artery
LCX: Circumflex artery
RCA: Right coronary artery
DS: Dysfunctional segments
